# Involvement of WNT Signaling in the Regulation of Gestational Age-Dependent Umbilical Cord-Derived Mesenchymal Stem Cell Proliferation

**DOI:** 10.1155/2017/8749751

**Published:** 2017-09-12

**Authors:** Sota Iwatani, Akemi Shono, Makiko Yoshida, Keiji Yamana, Khin Kyae Mon Thwin, Jumpei Kuroda, Daisuke Kurokawa, Tsubasa Koda, Kosuke Nishida, Toshihiko Ikuta, Kazumichi Fujioka, Masami Mizobuchi, Mariko Taniguchi-Ikeda, Ichiro Morioka, Kazumoto Iijima, Noriyuki Nishimura

**Affiliations:** ^1^Department of Pediatrics, Kobe University Graduate School of Medicine, Kobe 6500017, Japan; ^2^Department of Pathology, Kobe Children's Hospital, Kobe 6500047, Japan; ^3^Department of Neonatology, Tokyo Metropolitan Children's Medical Center, Fuchu 1838561, Japan; ^4^Department of Pediatrics, Hyogo College of Medicine, Nishinomiya 6638181, Japan; ^5^Department of Developmental Pediatrics, Shizuoka Children's Hospital, Shizuoka 4208660, Japan

## Abstract

Mesenchymal stem cells (MSCs) are a heterogeneous cell population that is isolated initially from the bone marrow (BM) and subsequently almost all tissues including umbilical cord (UC). UC-derived MSCs (UC-MSCs) have attracted an increasing attention as a source for cell therapy against various degenerative diseases due to their vigorous proliferation and differentiation. Although the cell proliferation and differentiation of BM-derived MSCs is known to decline with age, the functional difference between preterm and term UC-MSCs is poorly characterized. In the present study, we isolated UC-MSCs from 23 infants delivered at 22–40 weeks of gestation and analyzed their gene expression and cell proliferation. Microarray analysis revealed that global gene expression in preterm UC-MSCs was distinct from term UC-MSCs. WNT signaling impacts on a variety of tissue stem cell proliferation and differentiation, and its pathway genes were enriched in differentially expressed genes between preterm and term UC-MSCs. Cell proliferation of preterm UC-MSCs was significantly enhanced compared to term UC-MSCs and counteracted by WNT signaling inhibitor XAV939. Furthermore, WNT2B expression in UC-MSCs showed a significant negative correlation with gestational age (GA). These results suggest that WNT signaling is involved in the regulation of GA-dependent UC-MSC proliferation.

## 1. Introduction

Mesenchymal stem cells (MSCs) are a heterogeneous cell population that has a potential to proliferate and differentiate into trilineage mesenchymal cells: adipocytes, osteocytes, and chondrocytes. MSCs were initially isolated and characterized from the bone marrow (BM) [1, 2] and subsequently derived from almost all tissues including adipose tissue (AT), synovium, skin, dental pulp, umbilical cord blood (UCB), placenta, and umbilical cord (UC) [3]. Due to the ability to home to sites of injury, undergo differentiation, suppress immune responses, and modulate angiogenesis, MSCs are paid an increasing attention as a source for cell therapy against various degenerative diseases. Currently, MSCs from different sources have been tested in clinical studies for treatment of graft-versus-host disease, myocardial infarction, cerebral infarction, and so on [4, 5].

Although BM is the most well-characterized source of MSCs, it has certain limitations with the invasive BM aspiration and the decline in MSC proliferation and differentiation capacity with age. In contrast, fetal MSCs obtained from UCB, placenta, and UC have advantages with the noninvasive sampling during newborn delivery and the vigorous proliferation and differentiation capacity for cell therapy [6, 7]. Especially, human UC starts to develop at 4–8 weeks of gestation, continues to grow until 50–60 cm in length, and is usually discarded as medical waste after newborn delivery. Taken together, UC-derived MSCs (UC-MSCs) will become a promising source for cell therapy [8, 9].

Various genes and signaling pathways are known to regulate MSC proliferation and differentiation. WNT signaling serves as a key regulator that influences various stages of embryonic development as well as tissue homeostasis in adulthood [10]. It affects the proliferation, self-renewal, and differentiation of various tissue stem cells and controls the various tissue renewal and regeneration in response to disease, trauma, and ageing [11]. WNT ligands, which comprise a family of 19 members in human, are evolutionally conserved, are lipid modified, and secreted glycoproteins. They can activate either *β*-catenin-dependent (canonical) or *β*-catenin-independent (noncanonical) pathways by acting on transmembrane receptor FZD and its coreceptors LRP5/LRP6. The canonical pathway inhibits the *β*-catenin destruction complex, associates the transcriptional coactivator *β*-catenin with the transcriptional factor complex TCF/LEF, and induces WNT target gene transcription. The noncanonical pathway is independent of *β*-catenin and mainly associates with Ca^2+^-dependent and JNK-dependent signaling pathways, which can impact on cell migration, cell polarity, and cytoskeletal organization. The molecular events occurring these noncanonical pathways are far less defined than the canonical pathway [12].

Early studies showed the profound impacts of WNT signaling on a variety of tissue stem cell proliferation and differentiation [13–15]. In MSCs, both stimulatory and inhibitory roles for WNT signaling in cell proliferation and differentiation into trilineage mesenchymal cells were documented [16]. The adipogenic differentiation of AT-derived MSCs (AT-MSCs) was inhibited by WNT signaling activation [17]. The potential of WNT signaling on osteogenic differentiation of MSCs was controversial, with both stimulatory and inhibitory effects being reported [18, 19]. An inhibitory effect of WNT signaling on chondrogenic differentiation was demonstrated in AT-MSCs [20].

Although UC can be obtained from a wide range of gestational age (GA) newborn as a result of preterm, term, and postterm delivery, their functional differences are poorly characterized [21]. An understanding of the molecular mechanisms controlling UC-MSC proliferation and differentiation is crucial to determining the drivers and effectors of the functional difference between different GA UC-MSCs as well as the most suitable use of UC-MSCs for cell therapy against degenerative diseases. In the present study, we isolated UC-MSCs from 23 infants delivered at 22–40 weeks of gestation and analyzed their gene expression and cell proliferation.

## 2. Materials and Methods

### 2.1. Patients and Samples

Human UCs were obtained from 23 infants delivered at 22–40 weeks of gestation with parental written consent. This study was approved by the Ethics Committee at Kobe University Graduate School of Medicine (approval number 1370) and Hyogo Prefectural Kobe Children's Hospital (approval numbers 24-25) and conducted in accordance with the approved guidelines.

### 2.2. Preparation of UC-MSC

The umbilical cord (2-3 g weight) was collected, cut into 2-3 mm pieces, enzymatically dissociated with Liberase DH Research Grade (Roche, Mannheim, Germany) in PBS for 45–60 min at 37°C followed by the addition of 10% fetal bovine serum (FBS; Sigma, St. Louis, MO) to inhibit enzyme activity, and filtered through a 100 *μ*m cell strainer (BD Bioscience, Bedford, MA). The resulting cells derived from all compartments of the umbilical cord (whole UC) were cultured at 37°C (5% CO_2_ and 95% air) in MEM-*α* (Wako Pure Chemical, Osaka, Japan) containing 10% FBS and 1% antibiotic-antimycotic solution (Invitrogen, Carlsbad, CA) until confluent primary cultures were established. The cells were then disassociated with trypsin-EDTA (Wako Pure Chemical), and the trypsinized cells were seeded into fresh dishes and passaged to confluence. Serial passaging was carried out until the tenth passage. The cells at fifth to eighth passages were used in the present experiments.

### 2.3. Cell Surface Marker Analysis

UC-MSCs were dissociated with 0.25% trypsin-EDTA for 10 minutes, washed with PBS and suspended at ~1 × 10^6^ cells/ml in FCM buffer containing 1 × PBS, 2 mM EDTA, and 10% Block Ace (Dainippon Pharmaceutical, Osaka, Japan). The cells were incubated with phycoeryhrin- (PE-) conjugated mouse primary antibodies against CD14, CD19, CD34, CD45, CD73, CD90, CD105, or HLA-DR (BD Bioscience, Franklin Lakes, NJ) for 45 min on ice, washed with PBS, incubated with Fixable Viability Stain 450 (BD Bioscience) for 15 min at room temperature, washed with PBS, and filtered through a 70 *μ*m cell strainer (BD Bioscience). PE-conjugated mouse IgG1 k, IgG2a k, or IgG2b k isotype control (BD Bioscience) was used as a negative control for each primary antibody. Flow cytometric analysis was performed using FACSAria III carrying a triple laser (BD Bioscience) and FACSDiva software (BD Bioscience).

### 2.4. Cell Differentiation

To verify the multipotency of UC-MSCs, the cells were induced to differentiate into the adipogenic, osteogenic, and chondrogenic lineages. Adipogenic differentiation was induced in STEMPRO adipogenesis differentiation medium (Invitrogen) for 2-3 weeks and stained, and the differentiation was investigated by staining lipid vesicles with Oil Red O (Sigma). Osteogenic differentiation was induced in STEMPRO osteogenesis differentiation medium (Invitrogen) or STK-3 (DS Pharma Biomedical, Osaka, Japan) for 1-2 weeks, and the differentiation was examined by staining with Arizarin Red S (Sigma) reacting to calcium cation. Chondrogenic differentiation was induced by forming cell aggregates in micromass culture in STEMPRO chondorogenesis differentiation medium (Invitrogen) for 1 week, and the differentiation was assessed by staining anionic glycoconjugates with Toluidine Blue (Sigma). Cell images were acquired using a BZ-X700 microscope (Keyence, Osaka, Japan).

### 2.5. RNA Extraction

Total RNA from UC-MSCs and fibroblasts was extracted with a TRIZOL Plus RNA purification kit (Life Technologies) according to the manufacturer's instructions. RNA integrity was evaluated by Agilent 2100 Bioanalyzer (Agilent Technologies, Santa Clara, CA) using RNA 6000 nanokit (Agilent Technologies) according to the manufacturer's instructions.

### 2.6. Gene Expression Microarray Analysis

Total RNA from three term and five preterm UC-MSCs (Table 1) was subjected to global gene expression analysis using the Low Input Quick Amp Labeling Kit One-Color (Agilent Technologies) and SurePrint G3 Human Gene Expression v3 8 × 60 K Microarray Kit (Agilent Technologies) according to the manufacturer's instruction. Briefly, double-stranded cDNA was synthesized from 100 ng of total RNA by AffinityScript-RT using T7 promoter-incorporated Oligo-dT primer. Cyanine 3- (Cy3-) CTP-incorporated RNA (cRNA) was generated using the second strand cDNA as a template via an *in vitro* transcription reaction. The amplified cRNA was purified with the RNeasy mini kit (Qiagen, Valencia, CA) and quantified cRNA by the NanoDrop 2000 (Thermo Fisher Scientific, Waltham, MA). 600 ng of Cy3-labeled cRNA was hybridized to the microarray slides at 65°C for 17 hr with rotation at 10 rpm. After hybridization, the slides were washed and scanned by the SureScan (Agilent Technologies), the images were subsequently extracted using the Feature Extraction Software (Agilent Technologies). Extracted data with good QC metrics were normalized (percentile shift to the 75th percentile) and filtered by gene expression (20.0–100.0 percentile), flags for signals and error for CV in the GeneSpring GX (v 14.5) (Agilent Technologies). The processed data were subjected to statistical analysis (moderated *T*-test with Benjamini-Hochberg FDR), and the corrected *p* value <0.05 was determined to be significant (*n* = 3–5). The following analyses were performed for further data interpretation: principal component analysis (PCA), clustering analysis, GO (gene ontology) analysis, and pathway analysis with curated datasets of WikiPathways (413 pathways) and KEGG (10 pathways). A gene-set list associated with human WNT signaling pathway (150 genes, 04310 from KEGG pathways) was obtained from a public database (https://www.stemformatics.org/).

### 2.7. Quantitative RT-PCR (RT-qPCR)

cDNA was synthesized from 1 *μ*g of total RNA from UC-MSCs by using a QuantiTect reverse transcription kit (Qiagen). Real-time PCR analysis was performed with an ABI 7500 real-time PCR system (Applied Biosystems, Foster City, CA) using FastStart Universal SYBR Green master mix (Roche) with 0.5 *μ*M sense and antisense primers and cDNA (corresponding to 12.5 ng total RNA) according to the manufacturer's instructions. Each cDNA was amplified with a precycling hold at 95°C for 10 min, followed by 40 cycles at 95°C for 15 sec and 60°C for 60 sec, and one cycle at 95°C for 15 sec, 60°C for 60 sec, 95°C for 15 sec, and 60°C for 15 sec. Relative expression of each transcript was calculated based on the ΔΔCt method using *β*-actin (ACTB) as an endogenous reference for normalization. Primer sequences for WNT2, WNT2B, WNT3A, WNT4, WNT5B, WNT6, SFRP1, and ACTB were shown in Table 2. All sample measurements were repeated at least three times, and the results were expressed as the mean ± SE.

### 2.8. Ki-67 Staining

Cell suspensions of UC-MSCs were centrifuged at 3000 rpm for 5 min, and two smears were immediately prepared. Slides were fixed in 95% ethanol for immunostaining or fixed in 20% formalin and 80% methanol and stained with hematoxylin and eosin (H&E), respectively. Immunostaining was performed with antibody against Ki-67 (Clone MIB-1, Dako, Santa Clara, CA) using Leica Bond-Max automation and Bond Polymer Refine detection kit (Leica Biosystems, Nussloch, Germany) according to manufacturer's instructions. IHC cytology protocol included primary antibody incubation for 15 min, post primary for 8 min, polymer for 8 min, peroxide block for 5 min, mixed DAB refine for 10 min, and followed by 5 min hematoxylin counterstaining.

### 2.9. MTS Assay

UC-MSCs were seeded at the density of 12,000 cells/well in a 12-well plate, incubated in 1 ml of MEM-*α* with 10% FBS in the presence or absence of 10 *μ*M XAV939 (Selleck Chemicals, Houston, TX) at 37°C (5% CO_2_ and 95% air) for 24, 48, or 72 h. Cell proliferation was then determined by the CellTiter 96H AQueous One Solution Cell Proliferation Assay kit (Promega, Madison, WI, USA) according to the manufacturer's instruction. Briefly, 200 *μ*l of MTS reagent (a tetrazolium compound) was added into each well and incubated at 37°C (5% CO_2_ and 95% air) for 4 h. The absorbance at 490 nm was measured using an EnSpire Microplate Reader (Perkin Elmer, Poland, OR). All experiments were repeated at least three times, and the results were expressed as the mean ± SE.

### 2.10. Statistical Analysis

Pearson's correlation coefficients were determined, and the Mann–Whitney *U* test was used to compare two independent datasets, using Excel software (Microsoft, Redmond, WA) and Excel Statistics (Statcel 3; Social Survey Research Information, Tokyo, Japan). Differences were considered statistically significant for *p* < 0.05.

## 3. Results

### 3.1. UC-MSCs Isolated from Infants Delivered at 22–40 Weeks of Gestation

**Figure 1:**
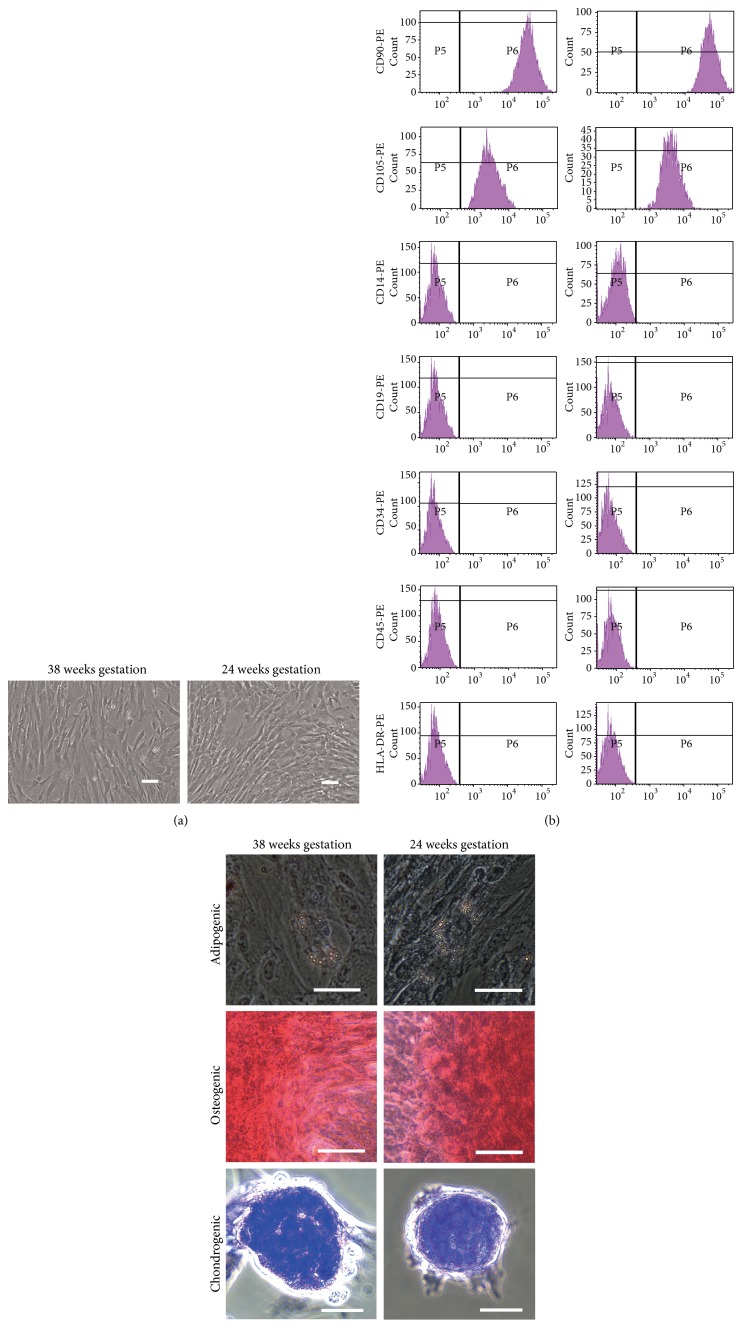
Characterization of UC-MSCs from term and preterm infants. (a) UC-MSCs from preterm (24 weeks of gestation, preterm UC-MSCs) and term (38 weeks of gestation, term UC-MSCs) newborns at passage numbers 6 to 7 were examined by phase-contrast microscopy. The images shown are representative of three independent experiments. Scale bars show 100 *μ*m. (b) Preterm and term UC-MSCs were analyzed by flow cytometer using antibodies against MSC markers (CD14, CD19, CD34, CD45, CD73, CD90, CD105, and HLA-DR) defined by ISCT [22]. The histograms shown are representative of three independent experiments. (c) Preterm and term UC-MSCs were differentiated into adipocyte as visualized by Oil Red O and into osteocyte as visualized by Alizarin Red S and chondrocyte as visualized by Toluidine Blue. The images shown are representative of three independent experiments. Scale bars represent 50 *μ*m.

We first obtained UCs from infants delivered at 22–40 weeks of gestation and then isolated the plastic-adherent cells from these UCs (Table 1). The cells exhibited a spindle-like shape (Figure 1(a)). Their cell surface markers were positive for MSC signature markers CD73, CD90, and CD105 but negative for hematopoietic, macrophage, and endothelial markers CD14, CD19, CD34, CD45, and HLA-DR by flow cytometric analysis (Figure 1(b)). There were no statistically significant differences in the percentages of MSC signature marker-positive cells (CD73: 99.9 ± 0.1% and 99.6 ± 0.4%, CD90: 99.9 ± 0.1% and 99.5 ± 0.5%, and CD105: 99.7 ± 0.3% and 99.5 ± 0.5%) between preterm and term UCs.

Under standard *in vitro* differentiation conditions, both preterm and term UC-MSCs were induced to differentiate into osteocytes, adipocytes, and chondrocytes (Figure 1(c)). Preterm UC-MSCs did not qualitatively differ from term UC-MSCs in their capacity to differentiate into trilineage mesenchymal cells. Taken together, the resulting cells fulfilled the criteria defined by the ISCT position paper [22] and were defined as UC-MSCs.

### 3.2. Differentially Expressed Genes between Preterm and Term UC-MSCs

**Figure 2:**
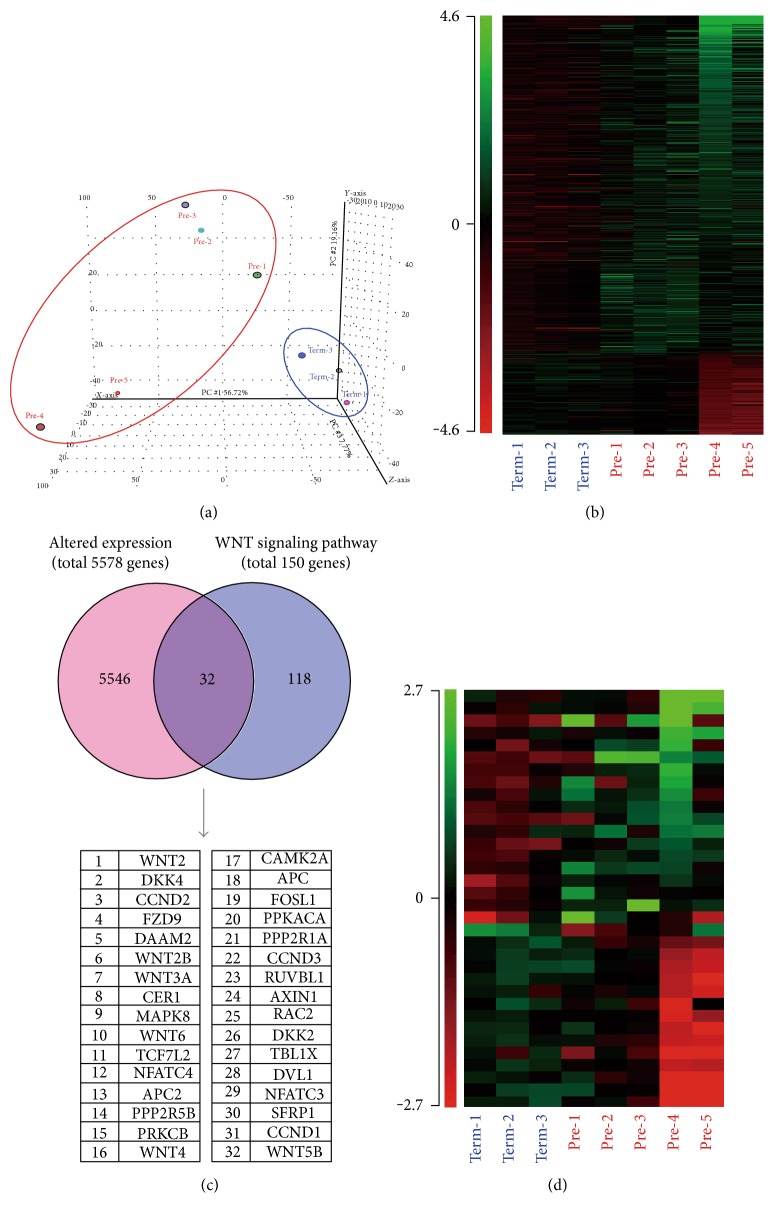
Gene expression microarray analysis of preterm and term UC-MSCs. (a) PCA mapping of gene expression profile for preterm (pre-1-5) and term (term-1-3) UC-MSCs. (b) Heat map of 5578 differentially expressed gene with greater than twofold changes in preterm UC-MSCs as compared to term UC-MSCs at a corrected *p* value less than 0.05. Green color refers to low levels of gene expression and red color to high levels. (c) Pie chart of altered expression genes and WNT signaling pathway genes. The list shows overlapped 32 genes. (d) Heat map of 32 genes extracted from (c). Green color refers to low levels of gene expression and red color to high levels.

To get an insight into the functional difference between preterm and term UC-MSCs, we extracted total RNA from five preterm and three term UC-MSCs (Table 1) and performed microarray analysis. Principal component analysis (PCA) for global gene expression revealed that preterm UC-MSC samples were clustered together and were separated from term UC-MSC samples (Figure 2(a)). In total, 5578 unique genes (4272 upregulated and 1306 downregulated) showed greater than twofold-expression changes between preterm and term UC-MSCs with a corrected *p* value less than 0.05 (Figure 2(b), Supplementary Table S1 available online at https://doi.org/10.1155/2017/8749751). The pathway analysis of all differentially expressed genes identified significant enrichment of signaling pathways for immune/inflammatory reactions, cell-cell/cell-extracellular matrix interactions, glucose/lipid metabolism, and cell proliferation and differentiation (Table 3). Among these signaling pathways, we focused WNT signaling pathway that was previously implicated in the regulation of MSC proliferation and differentiation. Noticeably, 32/150 of WNT signaling pathway genes were overlapped with differentially expressed genes between preterm and term UC-MSCs (Figures 2(c) and 2(d)).

**Figure 3:**
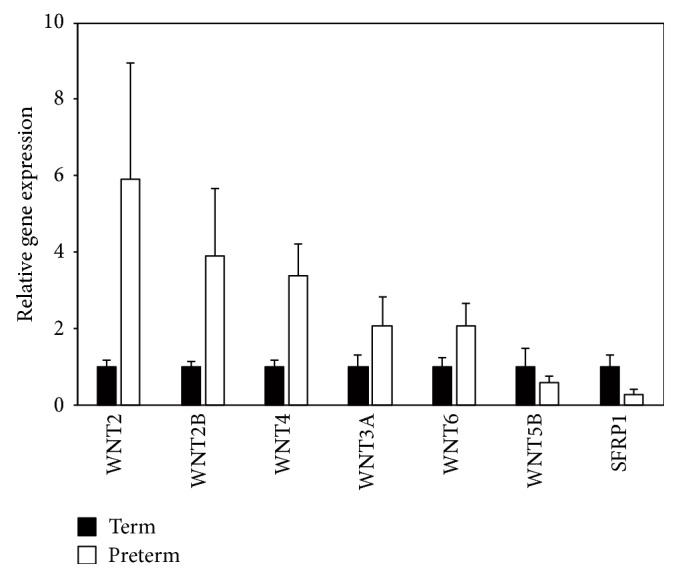
WNT signaling pathway gene expression in preterm and term UC-MSCs. The relative expression of WNT2, WNT2B, WNT3A, WNT4, WNT6, WNT5B, and SFRP1 mRNA in preterm (*n* = 3) and term (*n* = 5) UC-MSCs was analyzed by RT-qPCR. The mean of term UC-MSCs was set as 1. The results shown are the mean ± SE.

We then confirmed a subset of these WNT signaling pathway gene expressions by RT-qPCR using cDNA from the same five preterm and three term UC-MSCs as a template. A subset included secreted WNT ligands and modulators: WNT2, WNT2B, WNT3A, WNT4, WNT5B, WNT6, and SFRP1. Consistent with microarray analysis, upregulated WNT2, WNT2B, WNT3A, WNT4, and WNT6 showed increased expression in preterm UC-MSCs compared to term UC-MSCs by RT-qPCR (Table 4, Figure 3). Decreased expression of downregulated WNT5B and SFRP1 was also detected by RT-qPCR (Table 4, Figure 3). Collectively, these results suggested that WNT signaling pathway gene expression in preterm UC-MSCs was distinct from term UC-MSCs.

### 3.3. Cell Proliferation of Preterm and Term UC-MSCs

**Figure 4:**
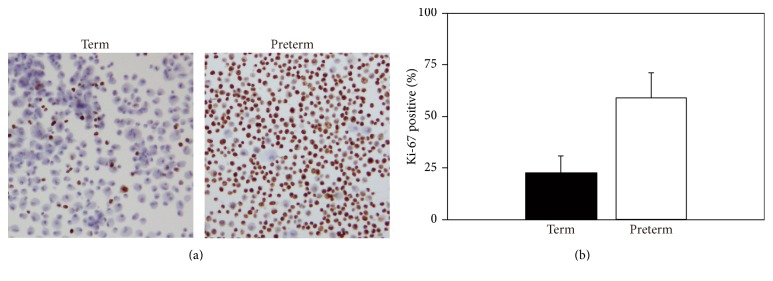
Ki-67 staining of preterm and term UC-MSCs. (a) Smears of preterm (*n* = 3) and term (*n* = 3) UC-MSCs were prepared, immunostained with anti-Ki-67 antibody, and counterstained with hematoxylin. The images shown are representative of three independent experiments. (b) The percentage of Ki-67 positive was determined by manually counting 1000 cells and expressed as the mean ± SE.

To examine the function of WNT signaling pathway genes in preterm and term UC-MSCs, we isolated UC-MSCs from nine preterm (22–26 weeks of gestation) and nine term (37–39 weeks of gestation) infants (Table 1) and analyzed their cell proliferation. We first evaluated the expression of Ki-67, a marker of proliferating cells expressed in all active phases of the cell cycle (G1, S, G2, and M), by immunocytochemistry [23]. The percentages of Ki-67-positive cells were markedly increased in preterm UC-MSCs as compared to term UC-MSCs, albeit not statistically significant (Figure 4).

**Figure 5:**
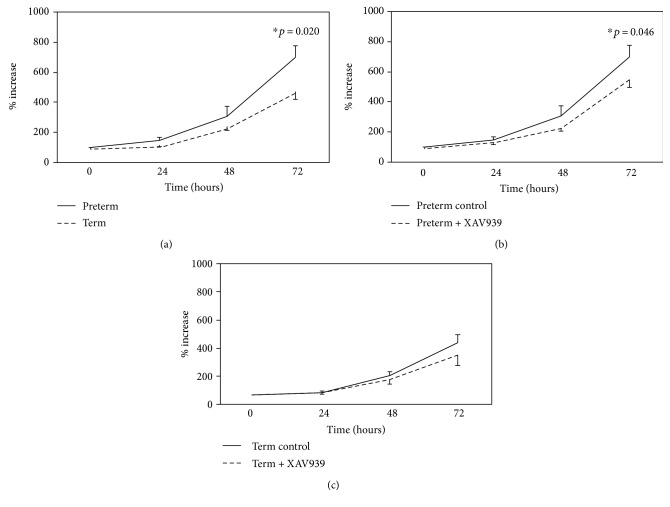
Cell proliferation of preterm and term UC-MSCs. Preterm (*n* = 8) and term (*n* = 6) UC-MSCs were cultured in the absence or presence of XAV939 for 24, 48, and 72 h. Their cell proliferation was determined by MTS assay and expressed as the percent increase. The results shown are the mean ± SE of (a) preterm and term UC-MSCs, (b) preterm UC-MSCs ± XAV939, and (c) term UC-MSCs ± XAV939.

We then analyzed cell proliferation of preterm and term UC-MSCs by MTS assay. Although both preterm and term UC-MSCs showed vigorous proliferation, the proliferation rate of preterm UC-MSCs measured at 72 h was significantly faster than term UC-MSCs (Figure 5(a)). Next, we examined the effect of WNT signaling inhibition on the growth of preterm and term UC-MSCs using a small molecule XAV939. XAV939 is a potent inhibitor of Tankyrase1 and Tankyrase2, and this inhibition stabilizes Axin1 and Axin2, the concentration-limiting component of the WNT pathway transcription factor *β*-catenin destruction complex. Increased levels of Axin1 and Axin2 stimulate *β*-catenin degradation and thereby inhibit *β*-catenin-mediated transcription [24]. Treatment of preterm UC-MSCs with 10 *μ*M XAV939 resulted in significant inhibition of cell proliferation (Figure 5(b)). Term UC-MSC proliferation was also reduced by 10 *μ*M XAV939, but there was no statistical significance (Figure 5(c)). These results suggest that WNT signaling is involved in the enhanced cell proliferation of preterm UC-MSCs compared to term UC-MSCs.

### 3.4. Gestational Age-Dependent Expression of WNT Signaling Pathway Genes

**Figure 6:**
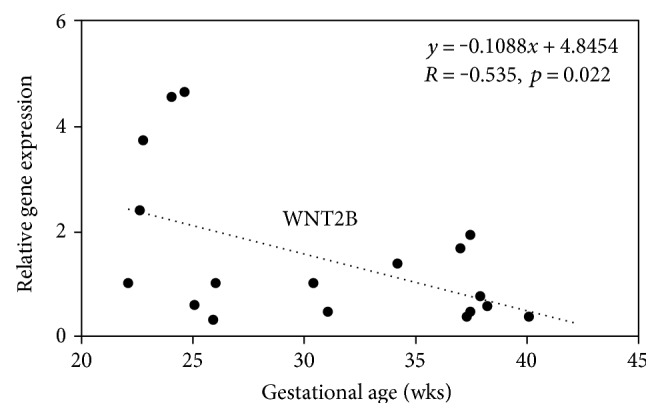
Gestational age-dependent expression of WNT signaling pathway genes. The relative expression of WNT2B mRNA in UC-MSCs isolated from 18 infants delivered at 22–40 weeks of gestation was analyzed by RT-qPCR. The mean of all UC-MSCs was defined as 1.

We further analyzed WNT2, WNT2B, WNT3A, WNT4, WNT5B, WNT6, and SFRP1 expressions in UC-MSCs isolated from other 10 infants delivered at 22–40 weeks of gestation by RT-qPCR. Expression of these WNT signaling pathway genes tended to decrease or increase with gestational age. Among them, WNT2B expression showed a statistically significant negative correlation with gestational age (Figure 6, Supplementary Figure S1).

## 4. Discussion

In the present study, we isolated UC-MSCs from 23 infants delivered at 22–40 weeks of gestation and obtained the following findings. (1) Global gene expression in preterm UC-MSCs was distinct from term UC-MSCs. (2) WNT signaling pathway genes were enriched in differentially expressed genes between preterm and term UC-MSCs. (3) Preterm UC-MSC proliferation was faster than term UC-MSCs. (4) WNT signaling inhibitor XAV939 significantly inhibited the cell proliferation of preterm but not term UC-MSCs. (5) WNT2B expression in UC-MSCs showed a significant negative correlation with GA.

MSCs are isolated from a variety of tissues and result in so heterogeneous population of cells, and not all of them express the same phenotypic markers. In the case of BM-MSCs, younger donor-derived BM-MSCs showed greater proliferative and differentiative potential than older counterparts and may have more potential for cell therapy [25, 26]. Although fetal MSCs could be isolated from newborns delivered at a wide range of GA as a result of preterm, term, and postterm delivery, their GA-dependent function remained poorly characterized [8, 9]. With regard to UCB-MSCs, the MSC population in UCB was significantly higher in preterm newborn compared to term newborn [27, 28]. In the case of UC-MSCs, MSCs were isolated from different UC compartments including cord lining, perivascular region (PV), Wharton's jelly (WJ), and whole UC [29–31]. Preterm UCs were shown to contain more perivascular cells (PVCs), identical to MSCs, than term UCs [32]. Preterm PVCs/UC-MSCs isolated from fetuses aborted at 8–12 weeks of gestation were reported to exhibit a greater proliferative potential, a more efficient differentiation into chondrogenic and adipogenic cell lineages, and a differential gene expression profile compared to term PVCs/UC-MSCs isolated from newborns delivered at 37–40 weeks of gestation [33]. Although we isolated UC-MSCs from the whole UC and preterm newborns delivered at 22–26 weeks of gestation, the present study and others supported that proliferative capacity of UC-MSCs declined with GA.

Global gene expression analysis identified 5578 differentially expressed genes between preterm and term UC-MSCs (Figure 2(a), Table S1). The pathway analysis revealed significant enrichment of 111 signaling pathways (Table 3). Immune/inflammatory reaction-associated signaling pathways were top-ranked among the list (Table 3). The upregulation of interferon (IFN) signaling pathways in preterm UC-MSCs may be interpreted as the consequence of preterm delivery that has inherent fetal and/or maternal indications (Table 1, Table 3). Although cell cycle and senescence-associated secretory phenotype pathways were also expected to affect the growth rate and GA-dependent changes of UC-MSCs, these pathways were not included in the list (Table 3).

WNT signaling is a key regulator of stem cell functions in development, renewal, and regeneration of multiple tissues [13–15]. In the case of MSCs, mRNA expression of a subset of WNT signaling pathway genes including WNT2, WNT4, WNT5A, WNT11, WNT16, SFRP2, SFRP3, and SFRP4 was detected in BM-MSCs [34]. WNT2, WNT2B, WNT4, WNT5A, WNT5B, SFRP1, and SFRP4 were also highly expressed in AT-MSCs under hypoxic stress conditions [35]. Comparison of BM-MSCs with UC-MSCs revealed lower differentiation capacity toward osteocytes and adipocytes along with the downregulation of WNT3A, WNT5A, WNT5B, WNT7B, WNT8A, SFRP1, and SFRP4 in UC-MSCs compared to BM-MSCs [36]. Consistent with these observations, the present study revealed a significant enrichment of WNT2, WNT2B, WNT3A, WNT4, WNT5B, WNT6, and SFRP1 in differentially expressed genes between preterm and term UC-MSCs (Figure 2(c)). Noticeably, WNT2, WNT2B, WNT4, WNT5B, WNT6, and SFRP1 were associated with a noncanonical WNT pathway, as opposed to only WNT3A with a canonical WNT pathway among these WNT ligands and modulators in UC-MSCs [12]. In contrast, the enhanced cell proliferation of preterm UC-MSCs was abolished by XAV939, which selectively decreased *β*-catenin expression through Tankyrase1 and Tankyrase2 inhibition and increased Axin1 and Axin2 expression (Figure 5) [24]. Accumulating evidence indicates that noncanonical WNT signaling can inhibit canonical WNT signaling [37, 38] and that activation of either canonical or noncanonical WNT signaling is highly dependent on the cell type and on specific receptors expressed by the cells [39, 40]. Further understanding of how WNT signaling pathway controls the GA-dependent proliferation of UC-MSC will be crucial to develop UC-MSC-based cell therapy.

In summary, preterm UC-MSC proliferation is significantly faster than term UC-MSCs, and WNT signaling is involved in the regulation of this GA-dependent proliferation of UC-MSCs.

## Supplementary Material

Table S1: List of differentially expressed genes between preterm and term UC-MSCs. Figure S1: Gestational age-dependent expression of WNT signaling pathway genes. Figure S1 legend: Relative expression of WNT2, WNT3A, WNT4, WNT5B, WNT6, and SFRP1 mRNA in UC-MSCs isolated from 18 infants delivered at 22–40 weeks of gestation was analyzed by RT-qPCR. The mean of all UC-MSCs was defined as 1.

## Figures and Tables

**Table 1:** UC-MSC sample characteristics.

Sample	Gestational age (weeks)	Birth weight (g)	Sex	Apgar score 1 min	Apgar score 5 min	Paternal age (years)	Maternal age (years)	Gravidity	Parity	Perinatal history	Maternal complication	Usage in the present study
Pre-1	24	530	Female	1	3	42	38	2	2	Cesarean section due to non-reassuring fetal status involved with abruption of placenta	Pregnancy-induced hypertension	Figures 2, 3, 5, and 6, S
Pre-2	25	656	Male	4	8	26	31	0	0	Cesarean section due to active premature labor		Figures 2, 3, 5, and 6, S
Pre-3	26	338	Male	2	2	37	36	1	1	Cesarean section due to active premature labor involved with placental hematoma		Figures 2, 3, 4, 5, and 6, S
Pre-4	22	550	Male	1	4	26	25	1	1	Cesarean section due to active premature labor		Figures 2, 3, 5, and 6, S
Pre-5	23	530	Female	2	4	32	31	1	1	Vaginal delivery due to active premature labor		Figures 2, 3, 5, and 6, S
Pre-6	23	478	Female	1	5	32	32	2	1	Cesarean section due to active premature labor		Figures 5 and 6, S
Pre-7	24	642	Male	1	3	16	17	0	0	Cesarean section due to active premature labor		Figures 1, 5, and 6, S
Pre-8	26	750	Female	5	9	39	38	2	1	Cesarean section due to advancing pregnancy-induced hypertension	Pregnancy-induced hypertension Graves' disease	Figures 4, 5, and 6, S
Pre-9	26	568	Female	4	7	38	36	2	1	Cesarean section due to non-reassuring fetal status	Low-lying placenta	Figure 4
Int-1	30	1546	Female	7	8	44	42	0	0	Cesarean section due to non-reassuring fetal status		Figure 6, S
Int-2	31	1170	Male	4	7	27	28	1	1	Cesarean section due to non-reassuring fetal status	Pregnancy-induced hypertension	Figure 6, S
Int-3	34	2062	Female	7	9	48	42	3	2	Cesarean section due to non-reassuring fetal status	Pregnancy-induced hypertension	Figure 6, S
Term-1	38	2550	Male	8	8	37	34	0	0	Cesarean section due to placental previa		Figures 2, 3, and 6, S
Term-2	40	2546	Male	2	6	40	44	1	1	Cesarean section due to non-reassuring fetal status		Figures 2, 3, and 6, S
Term-3	38	3314	Male	8	8	40	33	0	0	Cesarean section due to breech presentation		Figures 2, 3, 5, and 6, S
Term-4	37	2750	Female	8	9	36	33	0	0	Cesarean section due to placental previa		Figures 5 and 6, S
Term-5	37	3062	Male	9	10	35	35	1	1	Repeated cesarean section		Figure 5
Term-6	37	2776	Female	8	9	42	45	2	1	Repeated cesarean section		Figures 5 and 6, S
Term-7	38	3390	Male	9	10	32	26	1	1	Normal vaginal delivery		Figures 5 and 6, S
Term-8	38	2960	Male	8	9	38	39	1	1	Repeated cesarean section		Figures 1, 5, and 6, S
Term-9	38	3144	Female	8	8	39	39	1	1	Repeated cesarean section		Figure 4
Term-10	38	2666	Female	8	9	28	28	0	0	Cesarean section due to breech presentation		Figure 4
Term-11	39	2892	Female	9	9	37	31	1	0	Cesarean section due to breech presentation		Figure 4

**Table 2:** Primers used for RT-qPCR.

	Forward primer	Reverse primer
*WNT2*	tttggcagggtcctactcc	cctggtgatggcaaatacaa
*WNT2B*	aacttacataataaccgctgtggtc	actcacgccatggcactt
*WNT3A*	aactgcaccaccgtccac	aaggccgactccctggta
*WNT4*	gcagagccctcatgaacct	cacccgcatgtgtgtcag
*WNT5B*	gcgagaagactggaatcagg	cagagcagccgtgaacag
*WNT6*	agagtgccagttccagttcc	gaacacgaaggccgtctc
*SFRP1*	gctggagcacgagaccat	tggcagttcttgttgagca
*ACTB*	ccaaccgcgagaagatga	ccagaggcgtacagggatag

**Table 3:** Pathway analysis of differentially expressed genes between preterm and term UC-MSCs.

Pathway	*p* value	Matched entities	Pathway entities
Hs_Interferon_alpha-beta_signaling_WP1835_83224	1.91*E*−09	29	122
Hs_GPCR_downstream_signaling_WP1824_83301	1.85*E*−07	171	919
Hs_Interferon_gamma_signaling_WP1836_83234	8.41*E*−06	32	170
Hs_NRF2_pathway_WP2884_83041	3.20*E*−05	36	143
Hs_Immunoregulatory_interactions_between_a_Lymphoid_and_a_non-Lymphoid_cell_WP1829_83164	5.33*E*−05	32	332
Hs_Gastrin-CREB_signalling_pathway_via_PKC_and_MAPK_WP2664_83266	1.55*E*−04	42	180
Hs_Focal_Adhesion_WP306_80308	2.25*E*−04	45	191
Hs_GPCR_ligand_binding_WP1825_83346	2.99*E*−04	85	438
Hs_Regulation_of_beta-cell_development_WP3513_83407	3.98*E*−04	11	28
Hs_Glycerophospholipid_biosynthesis_WP2740_83341	4.59*E*−04	26	96
Hs_Allograft_Rejection_WP2328_78554	5.49*E*−04	24	100
Hs_Extracellular_matrix_organization_WP2703_83106	6.47*E*−04	22	78
Hs_Selenium_Micronutrient_Network_WP15_82705	6.56*E*−04	23	84
MAPK signaling pathway	7.53*E*−04	55	257
Hs_MicroRNAs_in_cardiomyocyte_hypertrophy_WP1544_75258	7.89*E*−04	23	104
Hs_DNA_Damage_Response_(only_ATM_dependent)_WP710_79974	8.29*E*−04	29	114
Hs_Parkin-Ubiquitin_Proteasomal_System_pathway_WP2359_72121	9.16*E*−04	20	73
Hs_Cell_surface_interactions_at_the_vascular_wall_WP1794_83824	0.0010894155	25	99
Hs_BDNF_signaling_pathway_WP2380_79953	0.0012996251	34	144
Hs_Nuclear_Receptors_Meta-Pathway_WP2882_83040	0.0013138579	62	318
Hs_Wnt_Signaling_Pathway_WP428_79854	0.0013306512	19	67
Hs_B_Cell_Receptor_Signaling_Pathway_WP23_79985	0.0014992601	25	98
Hs_MAPK_Signaling_Pathway_WP382_79951	0.0016350249	38	168
Hs_SIDS_Susceptibility_Pathways_WP706_80056	0.0017464098	37	166
Hs_O-linked_glycosylation_WP3315_83262	0.0020349505	25	104
Hs_NGF_signalling_via_TRKA_from_the_plasma_membrane_WP1873_83147	0.0023317037	20	77
Hs_Arachidonic_acid_metabolism_WP2650_83044	0.0026565776	15	53
Hs_Vitamin_B12_Metabolism_WP1533_82707	0.0026565776	15	53
Hs_Folate_Metabolism_WP176_82704	0.0028243104	18	67
Hs_Nanoparticle-mediated_activation_of_receptor_signaling_WP2643_74251	0.0033263897	10	28
Hs_Muscle_contraction_WP1864_83290	0.0033954314	17	63
Hs_NCAM_signaling_for_neurite_out-growth_WP1866_83314	0.0033983262	12	40
Hs_Corticotropin-releasing_hormone_WP2355_79973	0.0034443145	23	92
Hs_Cori_Cycle_WP1946_79691	0.0037244426	7	23
Hs_Eicosanoid_Synthesis_WP167_82702	0.0037961914	8	25
Hs_Serotonin_Receptor_2_and_ELK-SRF-GATA4_signaling_WP732_80010	0.0037961914	8	20
Hs_Semaphorin_interactions_WP1907_83271	0.0040335725	18	67
Hs_Histidine,_lysine,_phenylalanine,_tyrosine,_proline_and_tryptophan_catabolism_WP3573_83463	0.005489827	12	39
Hs_ACE_Inhibitor_Pathway_WP554_77712	0.0055746404	7	17
Hs_Integrin-mediated_Cell_Adhesion_WP185_80036	0.005693194	24	101
Hs_Prostaglandin_Synthesis_and_Regulation_WP98_72088	0.0058502043	10	31
Hs_GPCRs,_Class_A_Rhodopsin-like_WP455_81793	0.0062826765	50	262
Hs_Phase_1_-_Functionalization_of_compounds_WP1879_83057	0.0067225243	21	88
Hs_Complement_and_Coagulation_Cascades_WP558_79680	0.007009079	16	61
Hs_Lipid_digestion,_mobilization,_and_transport_WP2764_83187	0.0071218973	15	61
Hs_Type_II_diabetes_mellitus_WP1584_81779	0.0074452385	8	22
Hs_miRNA_targets_in_ECM_and_membrane_receptors_WP2911_83020	0.0074452385	8	45
Hs_Insulin_Signaling_WP481_82731	0.0084877005	34	161
Hs_L1CAM_interactions_WP1843_83082	0.008832967	21	92
Hs_Calcium_Regulation_in_the_Cardiac_Cell_WP536_80211	0.008975188	32	150
Hs_Cell_junction_organization_WP1793_83402	0.009355909	20	85
Hs_XBP1(S)_activates_chaperone_genes_WP3472_83243	0.010727612	22	98
Hs_G1_to_S_cell_cycle_control_WP45_80001	0.010931775	17	68
Hs_Prostate_Cancer_WP2263_80439	0.011036183	25	117
Hs_Wnt_Signaling_Pathway_and_Pluripotency_WP399_79474	0.011321301	23	101
Hs_Human_Complement_System_WP2806_83005	0.011321301	23	136
Hs_Assembly_of_collagen_fibrils_and_other_multimeric_structures_WP2798_83231	0.0113662705	9	29
Hs_DSCAM_interactions_WP1808_83372	0.0118092755	5	11
Hs_Arrhythmogenic_Right_Ventricular_Cardiomyopathy_WP2118_71265	0.012006286	18	78
Hs_Endothelin_Pathways_WP2197_74852	0.0121577475	10	33
Hs_Nuclear_Receptors_WP170_71083	0.012546546	11	38
Hs_Metapathway_biotransformation_WP702_73516	0.013431928	34	188
Hs_Potassium_Channels_WP2669_83272	0.0139101725	15	59
Hs_Myometrial_Relaxation_and_Contraction_Pathways_WP289_81078	0.014395579	32	156
Hs_Collagen_biosynthesis_and_modifying_enzymes_WP2725_83130	0.014458477	14	54
Hs_Cardiac_Hypertrophic_Response_WP2795_78544	0.014458477	14	54
Hs_IL-3_Signaling_Pathway_WP286_78583	0.014911717	13	49
Hs_Hematopoietic_Stem_Cell_Differentiation_WP2849_83039	0.015318828	11	98
Hs_Telomere_Maintenance_WP1928_83097	0.016210536	15	61
Hs_Signaling_Pathways_in_Glioblastoma_WP2261_81197	0.016799	19	83
Glycolysis/Gluconeogenesis	0.017775405	16	66
Hs_Reversible_hydration_of_carbon_dioxide_WP2770_83176	0.017944276	5	12
Hs_Transport_of_inorganic_cations-anions_and_amino_acids-oligopeptides_WP1936_83267	0.01851932	21	97
Hs_Neural_Crest_Differentiation_WP2064_79263	0.01922032	22	101
Hs_Signaling_by_the_B_Cell_Receptor_(BCR)_WP2746_83158	0.020346014	24	247
Hs_Adipogenesis_WP236_80209	0.02145827	27	131
Hs_Secretion_of_Hydrochloric_Acid_in_Parietal_Cells_WP2597_78485	0.021901488	3	5
Pathways in cancer	0.023056423	59	327
Hs_NLR_Proteins_WP288_80026	0.02675793	4	10
Hs_Neurotransmitter_Release_Cycle_WP1871_83254	0.027170882	10	39
Hs_Transport_of_vitamins,_nucleosides,_and_related_molecules_WP1937_83207	0.027170882	10	38
Hs_S_Phase_WP2772_83395	0.02769413	25	123
Hs_Integrin_cell_surface_interactions_WP1833_83181	0.028421601	15	66
Hs_GABA_synthesis,_release,_reuptake_and_degradation_WP2685_83090	0.030757299	6	19
Hs_Transport_of_glucose_and_other_sugars,_bile_salts_and_organic_acids,_metal_ions_and_amine_compounds_WP1935_83132	0.0316663	21	100
Hs_Class_I_MHC_mediated_antigen_processing_&_presentation_WP3577_83467	0.032386538	49	330
Hs_Formation_of_Fibrin_Clot_(Clotting_Cascade)_WP1818_83143	0.032393858	10	39
Hs_Parkinsons_Disease_Pathway_WP2371_79766	0.032393858	10	71
Hs_ErbB_Signaling_Pathway_WP673_80202	0.032395784	13	55
Hs_Interferon_type_I_signaling_pathways_WP585_80201	0.032395784	13	54
Hs_Metabolism_of_water-soluble_vitamins_and_cofactors_WP1857_83083	0.033578653	19	93
Hs_Primary_Focal_Segmental_Glomerulosclerosis_FSGS_WP2572_79947	0.033947315	16	74
Hs_Glycolysis_and_Gluconeogenesis_WP534_78585	0.034427498	12	49
Hs_Interleukin-11_Signaling_Pathway_WP2332_79525	0.03641972	11	44
Hs_Sleep_regulation_WP3591_83861	0.0382818	10	39
Hs_Regulation_of_toll-like_receptor_signaling_pathway_WP1449_81172	0.03940498	27	149
Hs_PI_Metabolism_WP2747_83160	0.03972961	12	51
Hs_Signal_regulatory_protein_(SIRP)_family_interactions_WP1909_83190	0.0397491	4	11
Hs_Neurotoxicity_of_clostridium_toxins_WP2665_83321	0.0397491	4	22
Hs_Overview_of_nanoparticle_effects_WP3287_82926	0.039779507	6	22
Hs_Protein_folding_WP1892_83103	0.039869573	9	34
Hs_Platelet_homeostasis_WP1885_83192	0.041275427	15	70
Hs_Synthesis_of_DNA_WP1925_83144	0.04189585	20	98
Hs_Pathogenic_Escherichia_coli_infection_WP2272_78594	0.042428713	13	64
Hs_Mitotic_G1-G1-S_phases_WP1858_83315	0.042929105	25	128
Hs_Synaptic_Vesicle_Pathway_WP2267_78595	0.04557775	12	51
Hs_Spinal_Cord_Injury_WP2431_80343	0.046727844	23	117
Hs_Gamma_carboxylation,_hypusine_formation_and_arylsulfatase_activation_WP2762_83388	0.04724196	9	36
Hs_RAF-MAP_kinase_cascade_WP2735_83142	0.047904383	36	216
Hs_Binding_and_Uptake_of_Ligands_by_Scavenger_Receptors_WP2784_83217	0.048846867	11	195
Hs_Extracellular_vesicle-mediated_signaling_in_recipient_cells_WP2870_79555	0.04923168	8	30

**Table 4:** Differentially expressed WNT pathway genes between preterm and term UC-MSCs.

Gene	FC (pre versus term)	*p* (Corr)
*Ligands*		
*WNT2*	4.07917	0.01631
*WNT2B*	2.64791	0.02698
*WNT3A*	2.56017	0.02584
*WNT6*	2.35531	0.00938
*WNT4*	2.11623	0.00510
*WNT5B*	−3.93533	0.01317
*Receptors*		
*FZD9*	3.47820	0.04035
*TCF7L2*	2.29222	0.01187
*Extracellular modulators*		
*DKK4*	3.79029	0.01143
*DKK2*	−2.65327	0.01720
*SFRP1*	−3.13336	0.00621
*Intracellular signaling molecules*		
*CCND2*	3.49892	0.02933
*DAAM2*	2.71289	0.00467
*CER1*	2.40160	0.03446
*MAPK8*	2.36091	0.00501
*NFATC4*	2.25687	0.01089
*APC2*	2.21774	0.04864
*PPP2R5B*	2.20956	0.00501
*PRKCB*	2.11780	0.00504
*CAMK2A*	2.03443	0.02825
*APC*	−2.01861	0.00578
*FOSL1*	−2.06872	0.03346
*PRKACA*	−2.16403	0.02705
*PPP2R1A*	−2.19719	0.04119
*CCND3*	−2.20727	0.01748
*RUVBL1*	−2.24427	0.02646
*AXIN1*	−2.47832	0.02670
*RAC2*	−2.51880	0.00902
*TBL1X*	−2.73244	0.01261
*DVL1*	−2.77299	0.00726
*NFATC3*	−2.82561	0.02238
*CCND1*	−3.28580	0.03436
