# Supplementary figures and images for: Involvement of WNT Signaling in the Regulation of Gestational Age-Dependent Umbilical Cord-Derived Mesenchymal Stem Cell Proliferation
Source: Stem Cells Int. 2017 Sep 12;2017:8749751. doi: 10.1155/2017/8749751 (PMC5613457; doi:10.1155/2017/8749751)

FIGURE S1

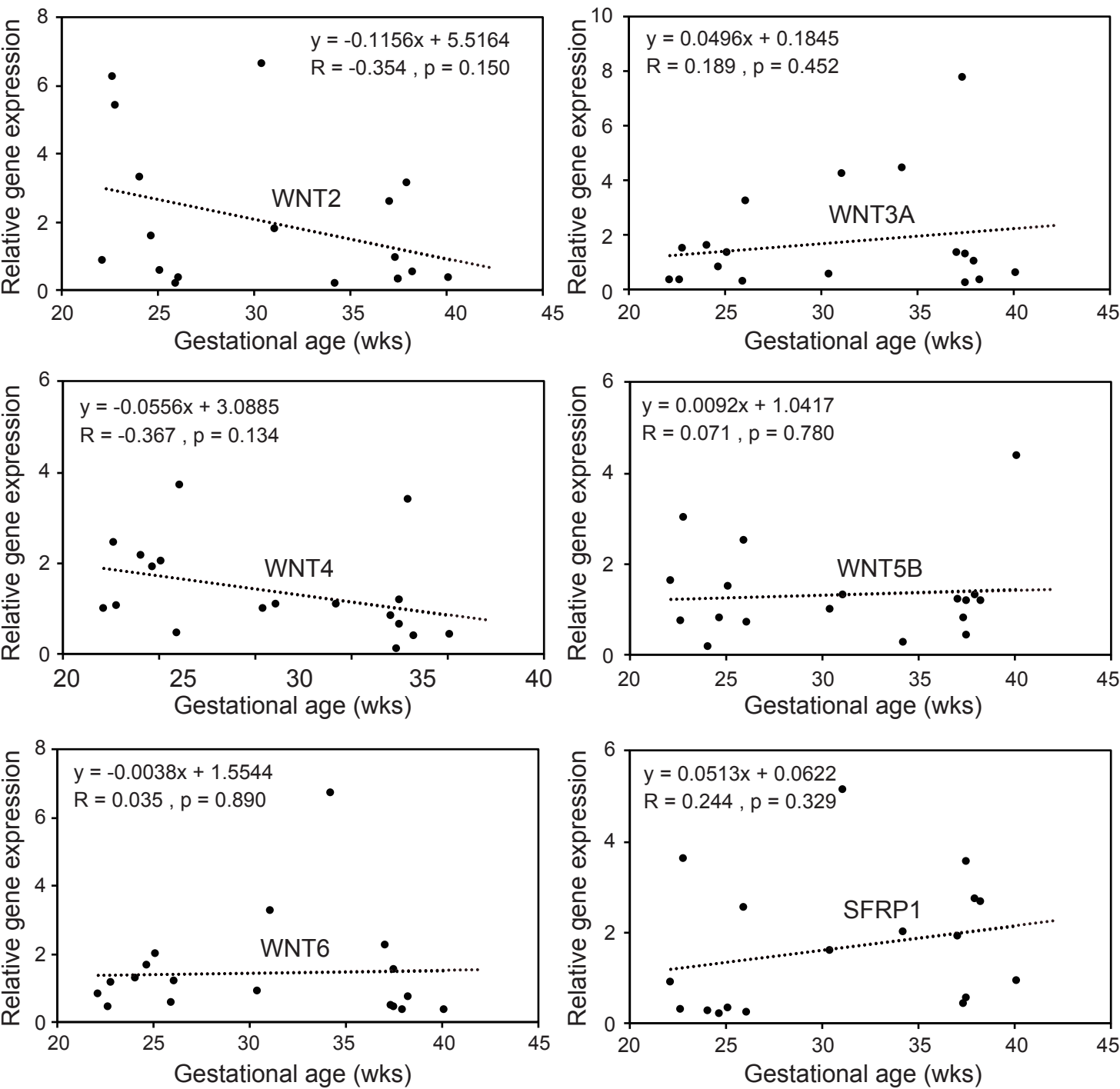

Supplement: Supplementary file 2 [file 8749751.f2.pdf]
